# Gastrointestinal Panel Performance for the Diagnosis of Acute Gastroenteritis in Pediatric Patients

**DOI:** 10.7759/cureus.61979

**Published:** 2024-06-08

**Authors:** Marwa Sameer, Abdulrahman Masood, Lateefa Almutawea, Gabriel Fox, Ramaning Loni, Amira Ahmed, Hadhami Ben Turkia, Maryam Abdulsamad, Imelda Mary

**Affiliations:** 1 Pediatric and Neonatology Department, King Hamad University Hospital, Muharraq, BHR; 2 Cardiology Department, Mohammed Bin Khalifa Bin Salman Al Khalifa Specialist Cardiac Center, Riffa, BHR; 3 Pathology, Blood Bank, and Laboratory Medicine Department, King Hamad University Hospital, Muharraq, BHR

**Keywords:** gastroenteritis, stool culture, stool analysis, bacterial and viral agents, enteric bacteria, gastrointestinal panel, acute diarrhea in children

## Abstract

Background: Various methods are used to identify the causative organisms of acute gastroenteritis (AGE) in children. The gastrointestinal (GI) panel has the potential to detect up to 22 pathogens rapidly through the multiplex real-time PCR test. We studied the impact of the GI panel on clinical management in the pediatric population.

Methods: A retrospective study was conducted to collect data on GI panel results and clinical details of inpatient children presenting with AGE at King Hamad University Hospital, Kingdom of Bahrain, over the course of one year.

Results: One hundred nine samples were collected. The GI panel was positive in 96 samples (88.1%), with the majority detected in the toddler age group. Forty-one (42.7%) samples were positive for at least one organism. *Salmonella* was the most frequently encountered bacteria as a single isolate, 10/55 (18.2%), while enteropathogenic *Escherichia coli* was the most common co-infected organism, 16/41 (39%). Norovirus was the most common virus among the viruses. Bacterial detection peaked from July to October, while viral detection plateaued throughout the year. The GI panel and stool culture were positive for the same organism in 17 samples, versus one sample with a different organism. Sixty-two (56.9%) samples had a positive GI panel but negative stool cultures and stool analysis, and half of those detected viruses. The GI panel was positive in 86.2% of severely ill patients; the majority were bacteria. Bacterial detection was associated with a higher CRP compared to viruses.

Conclusion: The GI panel is an informative tool for detecting the causative pathogen of AGE in children. However, it can detect multiple organisms, indicating a possible carrier status, which points toward future studies.

## Introduction

Pediatric diarrheal disease is a significant health-associated burden, both in developed as well as low-income developing countries, showing a high incidence worldwide, reaching 1.7 billion pediatric cases per year [1]. Although diarrheal-associated mortality has been markedly reduced globally, it is still considered to be responsible for half a million deaths per year in children less than five years of age [1,2]. The most common organisms, like viruses, bacteria, and parasites, are observed; however, noninfectious causes are also identified [3]. Rotavirus, *Salmonella*, and *Shigella* were reported as the most common causative organisms of severe gastroenteritis in pediatric patients in the Kingdom of Bahrain [4]. Early and accurate identification of a causative organism remains a principal factor influencing management, isolation, and antibiotic use, especially in pediatric patients [5-7]. Over the years, a significant focus has been on developing new methods to identify the various causative organisms of acute gastroenteritis (AGE). Conventional methods like stool culture remain the primary diagnostic method for suspected bacterial diarrhea; however, it is still considered time-consuming, labor-dependent, and restrictively sensitive [6]. On the other hand, the gastrointestinal (GI) panel approved by the FDA in 2014 is a promising method, testing for up to 22 gastroenteritis-causative organisms, including viruses, bacteria, and parasites, simplifying and accelerating reporting compared to conventional methods [2]. Our study aims to study the GI panel's performance in detecting the causative viral, bacterial, or parasitic organisms of AGE in children.

## Materials and methods

Patients and materials

This is a cross-sectional study conducted at King Hamad University Hospital, Bahrain, which is a tertiary-level government hospital catering to a population of more than 260,000. We retrospectively collected data on patients admitted to the pediatric wards, starting with the implementation of GI panel testing in April 2019 and continuing for 12 months until March 2020.

Inclusion and exclusion criteria

All pediatric patients below the age of 14 who were admitted to the pediatric ward or pediatric intensive care unit (PICU) for the management of AGE and whose stool samples were obtained for GI panel, stool analysis, and stool culture were included in the study.

Pediatric patients who are known to be immunocompromised and had prolonged hospital admissions due to chronic conditions or inadequate investigations were excluded from the study.

Data collection

The patient's data, such as the patient's age at presentation, gender, presenting symptoms, frequency of symptoms, hydration status, duration of hospital stay, and antibiotic use, were collected. The data were divided according to the age groups (neonates, infants, toddlers, pre-school, school age, and adolescents). Laboratory investigations, including WBC, CRP, stool analysis, stool cultures, and GI panel, were also collected. Severely ill patients were identified as patients who had moderate to severe dehydration, significantly elevated CRP (>50 mg/l), elevated lactate (>2.2 mmol/L) with metabolic acidosis, or required admission to the pediatric ICU.

Microbiology

The pediatric patients' diarrheal stool samples were sent to the microbiology lab for molecular testing using the Biofire GI panel (bioMérieux, Marcy-l'Étoile, France). This is a multiplexed nucleic acid test that can identify the nucleic acids of many bacteria, viruses, and parasites directly from stool samples collected in the Cary-Blair transport medium. The PCR panel allows the detection of bacterial agents (*Campylobacter jejuni*, *Clostridioides difficile*, *Plesiomonas shigelloides*, *Salmonella*, *Vibrio*, including *Vibrio cholerae*, *Vibrio parahaemolyticus*, *Vibrio vulnificus*, *Yersinia enterocolitica*, enteroaggregative *Escherichia coli* (EAEC), enteropathogenic *Escherichia coli* (EPEC), enterotoxigenic *Escherichia coli* (ETEC), Shiga-like toxin-producing *Escherichia coli* (STEC), including specific identification of the *Escherichia coli* O157 serogroup within STEC, *Shigella*/enteroinvasive *Escherichia coli* (EIEC)), viral agents (adenovirus F 40/41, astrovirus, norovirus, rotavirus A, and sapovirus), and protozoal agents (*Cryptosporidium*, *Cyclospora cayetanensis*, *Entamoeba histolytica*, *Giardia lamblia*). Detected organisms were reported through the hospital laboratory information system.

Ethical consideration

This study was conducted in accordance with the Helsinki Declaration; it was reviewed and approved by the Institutional Review Board at King Hamad University Hospital (approval number: 20-326). Informed consent was obtained from every parent or legal guardian upon admission.

Statistical analysis

Data were initially entered in an Excel sheet (Microsoft Corporation, Washington, USA) and then transferred to SPSS Statistics version 21.0 (IBM Corp. Released 2012. IBM SPSS Statistics for Windows, Version 21.0. Armonk, NY: IBM Corp.) for analysis.

## Results

A total of 220 stool samples from pediatric patients with AGE were collected, of whom 111 were excluded as they did not meet the inclusion criteria. Table 1 defines the characteristics of the final 109 samples enrolled in the study. Ninety-eight (88.1%) samples had positive GI panel results, while 13 (11.4%) were unremarkable. The total sample showed no significant difference in gender distribution. Ninety-six samples had a positive GI panel, of which 80% were younger than six years. The median age of the patients was 2.1 years (IQR, 11 months to 4.3 years). Toddlers and preschoolers were the most common age groups with a positive GI panel (32.4% and 29.3%, respectively).

**Table 1:** Characteristics of the 109 samples collected for pediatric patients diagnosed with AGE AGE: acute gastroenteritis

Patient’s characteristics	Total N (%)	Positive samples N (%)
Gender		
Male	54 (49.5%)	47 (49.0%)
Female	55 (50.5%)	49 (51.0%)
Age		
Neonates (<1 month)	4 (3.7%)	2 (2.1%)
Infants (1 month to <1 year)	25 (22.9%)	20 (20.8%)
Toddlers (1 year to <3 years)	34 (31.2%)	31 (32.4%)
Preschool (3 years to <6 years)	30 (27.5%)	28 (29.3%)
School-age (6 years to <12 years)	12 (11.0%)	11 (11.5%)
Adolescents (>12 years)	4 (3.7%)	4 (4.2%)

Most patients presented with diarrhea, and more than 53% of them were identified as severely ill upon presentation; fever, vomiting, abdominal pain, and dehydration were variable among patients. Regardless of GI panel results, there was no significant difference in clinical severity or rate of pediatric intensive care admission (Table 2).

**Table 2:** Clinical pictures of 109 samples tested for GI panel GI: gastrointestinal, PICU: pediatric intensive care unit

Clinical picture	Bacteria only	Virus only	Parasite only	Mixed	Negative	p-value
Fever						
Yes	51 (86.4%)	12 (80.0%)	0 (0.0%)	16 (76.2%)	10 (76.9%)	0.2
No	8 (13.6%)	3 (20.0%)	1 (100.0%)	5 (23.8%)	3 (23.1%)	
Vomiting						
Yes	41 (69.5%)	13 (86.7%)	1 (100.0%)	18 (85.7%)	8 (61.5%)	0.308
No	18 (30.5%)	2 (13.3%)	0 (0.0%)	3 (14.3%)	5 (38.5%)	
Diarrhea						
Yes	56 (94.9%)	14 (93.3%)	1 (100.0%)	21 (100.0%)	13 (100.0%)	0.727
No	3 (5.1%)	1 (6.7%)	0 (0.0%)	0 (0.0%)	0 (0.0%)	
Abdominal pain						
Yes	26 (44.1%)	3 (20.0%)	1 (100.0%)	6 (28.6%)	4 (30.8%)	0.223
No	33 (55.9%)	12 (80.0%)	0 (0.0%)	15 (71.4%)	9 (69.2%)	
Clinically sick						
Yes	33 (55.9%)	9 (60.0%)	0 (0.0%)	8 (38.1%)	8 (61.5%)	0.423
No	26 (44.1%)	6 (40.0%)	1 (100.0%)	13 (61.9%)	5 (38.5%)	
PICU admission						
Yes	3 (5.1%)	1 (6.7%)	0 (0.0%)	1 (4.8%)	1 (7.7%)	0.992
No	56 (94.9%)	14 (93.3%)	1 (100.0%)	20 (95.2%)	12 (92.3%)	

**Figure 1:**
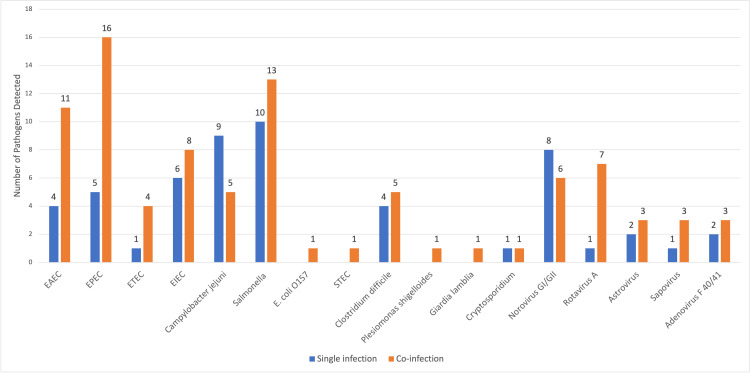
Number of organisms detected as a single infection and co-infection EAEC: enteroaggregative *Escherichia coli*, EPEC: enteropathogenic *Escherichia coli*, ETEC: enterotoxigenic *Escherichia coli*, EIEC: enteroinvasive *Escherichia coli*, STEC: Shiga-like toxin-producing *Escherichia coli*

The GI panel showed multiple organisms in 41 samples (42.7%) out of a total of 96 positive samples; 35/41 (85.3%) were positive for two organisms, and 6/41 (14.7%) were positive for three organisms. A single organism was detected in 55 of the total 96 positive samples (57.3%). An isolated bacteria was detected in 39/55 (70.90%), an isolated virus in 15/55 (27.27%), and an isolated parasite in 1/55 (1.81%). The most common organism detected as a single isolate was *Salmonella* in 10/55 (18.2%), followed by *Campylobacter jejuni* in 9/55 (16.4%), and norovirus in 8/55 (14.5%). Although *Salmonella* was the most commonly detected isolate, it was also found to be a co-infection in 13 out of 23 samples (56.5%). EPEC was most likely detected as a co-infection. *Cryptosporidium* and *Giardia lamblia* were isolated as a protozoal co-infection in two out of three samples (Figure 1).

**Figure 2:**
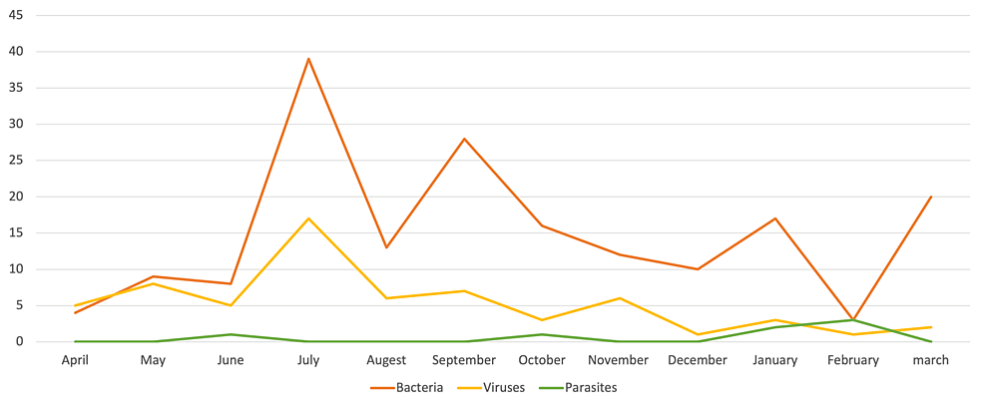
AGE causative organism distribution throughout the year AGE: acute gastroenteritis

Bacterial organisms were detected throughout the year, with a peak detection in the period from July to October and another smaller peak detected in January. Viruses were mostly noted to have plateaued in detection throughout the year (Figure 2).

Tables 3-5 display the three groups of organisms tested in the GI panel and their total distribution, numbers age-wise, with their corresponding average CRP level. Bacteria were more frequently isolated than viruses and parasites, representing 73.65% (N=104) of the isolates. *Salmonella*, EPEC, EAEC, EIEC, and *Campylobacter jejuni* were the most commonly detected bacterial organisms (Table 3). However, norovirus and rotavirus were the most prevalent viruses. Bacterial organisms were most common in toddlers as compared to viruses in infants. The mean CRP for all patients was 67.7 mg/l (mean ± SD = 67.7 ± 76.7). The CRP was significantly elevated in bacterial infections compared to viral or parasitic infections. The average CRP for EIEC was the highest at 147 mg/l (CRP range excluding three samples with a negative CRP: 85.4-326 mg/l). *Salmonella* samples had an average CRP of 100.19 mg/l (CRP range 10.5-309.4) (Table 3). The average CRP corresponding to sapovirus infection was found to be elevated (CRP 113.75 mg/l). However, sapovirus was mostly found to be co-detected. A co-detected sapovirus was associated with a higher CRP than a single isolate (Table 5, Figure 1).

**Table 3:** Bacterial detection according to age with corresponding average CRP CRP: C-reactive protein

Bacteria detected in the samples collected	Average CRP mg/l (82.03 mg/l)	Total number 104	Distribution according to age group (n)					
			Neonates	Infants	Toddlers	Pre-school	School-age	Adolescents
Enteroaggregative *Escherichia coli*	75.96	15	0	3	7	4	1	0
Enteropathogenic *Escherichia coli*	66.33	21	0	3	9	7	1	1
Enterotoxigenic *Escherichia coli*	34.2	5	0	1	3	1	0	0
Enteroinvasive *Escherichia coli*	147	14	0	1	2	6	4	1
Campylobacter jejuni	68.76	14	1	0	6	4	2	1
Salmonella	100.19	23	0	4	6	8	4	1
*Escherichia coli* O157	4.8	1	0	0	1	0	0	0
Shiga-like toxin-producing *Escherichia coli*	4.8	1	0	0	1	0	0	0
Clostridium difficile	44	9	0	4	4	1	0	0
Plesiomonas shigelloides	101	1	0	0	0	0	1	0
Vibrio cholerae	-	0	0	0	0	0	0	0
Yersinia enterocolitica	-	0	0	0	0	0	0	0

**Table 4:** Parasites detection according to age with corresponding average CRP CRP: C-reactive protein

Parasite detected in the samples collected	Average CRP mg/l (48.26 mg/l)	Total number (3)	Distribution according to age group (n)					
			Neonates	Infants	Toddlers	Pre-school	School-age	Adolescents
Cyclospora cayetanensis	-	0	0	0	0	0	0	0
Entamoeba histolytica	-	0	0	0	0	0	0	0
Giardia lamblia	124.2	1	0	0	0	1	0	0
Cryptosporidium	10.3	2	0	0	0	1	1	0

**Table 5:** Viral detection according to age with corresponding average CRP *Samples were co-detected with bacterial organisms that are commonly associated with high CRP (i.e., *Salmonella*, EIEC, and *Campylobacter*). CRP: C-reactive protein, EIEC: enteroinvasive *Escherichia coli*

Virus detected in the samples collected	Average CRP mg/l (33.68 mg/l)	Total number (34)	Distribution according to age group (n)					
			Neonates	Infants	Toddlers	Pre-school	School-age	Adolescents
Norovirus GI/GII	14.5	14	0	7	5	2	0	0
Rotavirus A	24.68	8	0	4	2	2	0	0
Astrovirus	46.82	5	0	0	1	3	0	1
Sapovirus*	113.75	4	0	0	2	1	0	1
Adenovirus	20.92	5	1	3	0	1	0	0

All patients had an average hospital stay of 4.5 days. There was no significant difference between the mean duration of hospitalization for bacterial and viral infections. When compared to the stool analysis and stool culture results, one sample showed a negative GI panel but a positive stool culture (*Salmonella*), and two samples showed a negative GI panel but an abnormal stool analysis. The GI panel and stool cultures were positive for the same organism in 16.6% of cases (N=17/96) but yielded different organisms in one patient; 14.6% (N=16/96) of GI panel samples also showed an abnormal stool analysis but a negative stool culture, and 62.4% (N=10/16) of these samples had an isolated EIEC. About 56.88% (N=62/96) had a positive GI panel with a simultaneous negative stool analysis and stool culture, out of which half (31/62, 50%) detected a virus. All three tests were positive in 8/109 (7.33%). All three were negative in 10/109 (9.2%).

A total of 58 GI panel samples were taken from clinically ill patients who presented with moderate to severe dehydration, significantly elevated CRP (>50 mg/l), elevated lactate (>2.2) with metabolic acidosis, or required admission to the PICU. Fifty out of 58 patients (86.20%) had a positive GI panel. Twenty-seven of 58 (46.55%) had a single positive GI panel, which included a negative stool analysis and stool culture. Most clinically ill patients were positive for bacteria alone (56.9%, p=0.036). There were no significant differences in age distribution when comparing GI panel results (chi-square p=0.048) and the clinical status of the patient (chi-square p=0.065) (Table 6).

**Table 6:** Age group distribution in relation to GI panel results, patient's clinical status, and type of infection

Category	Neonate	Infant	Toddler	Preschool	School age	Adolescent
GI panel						
Positive	2 (50%)	21 (80.8%)	31 (91.2%)	23 (100%)	15 (83.3%)	4 (100%)
Negative	2 (50%)	5 (19.2%)	3 (8.8%)	0 (0%)	3 (16.7%)	0 (0%)
Clinically sick						
Yes	3 (75%)	14 (53.8%)	12 (35.3%)	15 (65.2%)	13 (72.2%)	1 (25%)
No	1 (25%)	12 (46.2%)	22 (64.7%)	8 (34.8%)	5 (27.8%)	3 (75%)
Type of infection						
Bacterial	0 (0%)	3 (21.4%)	9 (75%)	12 (80%)	8 (61.5%)	1 (100%)
Viral	1 (33.3%)	5 (35.7%)	1 (8.3%)	1 (6.7%)	1 (7.7%)	0 (0%)
Mixed	0 (0%)	2 (14.3%)	2 (16.7%)	2 (13.3%)	2 (15.4%)	0 (0%)
Negative	2 (66.7%)	4 (28.6%)	0 (0%)	0 (0%)	2 (15.4%)	0 (0%)

## Discussion

This study evaluated the performance of the GI panel in pediatric patients who were admitted for AGE, which can rapidly detect a wide variety of bacteria, viruses, and parasites known to cause diarrheal illness. The GI panel demonstrated a higher positivity rate of 88.1% compared to similar previous studies [2,8,9]. A large, cross-sectional study with 8720 pediatric patients compared the GI panel to other common methods. The GI panel group had a 40% positivity rate [8], while Yoo et al. identified a higher positivity rate of 57.1% [2]. This might be explained by the higher rate of detection of non-viable pathogens and colonizers by the GI panel.

Children less than one year of age can have *Clostridium difficile* as a silent pathogen without clinical significance [9]. Symptomatic clostridial diarrhea in infants was rarely identified, despite an elevated colonization rate in this age group. The use of antibiotics, commonly third-generation cephalosporins, clindamycin, and amoxicillin-clavulanate, and an increased rate of healthcare facility visits showed significant risk factors for clostridial diarrhea in children [10].

EPEC was also found to be a prevalent organism identified in asymptomatic pediatric patients's stool samples, although it is still considered a major cause of AGE in children [11]. *Clostridium difficile* in infants and EPEC represented 24% of the organisms identified in our study; the latter was the most commonly co-infected. The rate of multiple organisms’ detection varied in previous similar studies; however, our study showed a higher rate (37.6%) [9,12]. This again increases the chance that the GI panel will find non-viable pathogens that aren't linked to diarrheal illness at the same time [13].

Our study's median age was 25 months, compared to 17 months in the United Arab Emirates (UAE) [12]. The studies conducted in neighboring countries (UAE and Qatar) demonstrated comparable results in that viruses were the most predominant causative pathogens, which is in contrast to our study that detected bacteria more predominantly, regardless if the sample detected one or multiple organisms [12,14] El-Tayeb et al. conducted a local study in 2019 that revealed an increased incidence of *Salmonella* infections among adolescents, while the incidence remained unchanged among younger age groups [15]. Our study identified *Salmonella* as the most common single-identified organism. Studies done in places where *Salmonella* is common suggested that chronic carrier status might be a reason for the high incidence. However, we could not find any information about how common chronic *Salmonella* and asymptomatic carriers are in Bahrain [16].

Norovirus was the most common virus detected by the GI panel, matching the results of recent similar studies [2,9,12]. Viral organism detection in our study contradicts the data given by Stockmann et al. in terms of seasonal variation, which showed frequent viral detection in the winter. On the other hand, it was agreed that bacterial infections predominate in the summer months [9]. Rotavirus was previously identified as a common cause of pediatric gastroenteritis hospitalization in Bahrain; however, it represented 7.7% of the total organisms detected in our study, which would be consistent with the rotavirus vaccination campaign in Bahrain [17].

Compared to viral and bacterial detection, the GI panel results in our study showed no impact on hospital stay length, suggesting that patients' symptoms primarily drive hospital stay length and that organism identification did not contribute to early discharge. Pinto et al. conducted a study to investigate the relationship between organism identification and length of stay, concluding that organism identification in patients with AGE did not ultimately shorten the length of stay [18].

Antibiotic use and management of bacterial GE were improved upon accelerated organism detection by the GI panel [2]. Empirical antibiotic therapy is considered in infants younger than three months of age in cases of fever with dysentery presumptive due to *Shigella* or *Salmonella* and/or a history of recent travel to endemic areas, but otherwise, it is not recommended [19]. The GI panel limited the unnecessary empirical therapy in GE where antibiotics were not indicated. Antibiotics were later stopped in 40% of our patients with GI panel-detecting viruses.

According to the guidelines of the Infectious Diseases Society of America, the GI panel is considered more sensitive but less dependent than conventional methods, despite its high sensitivity in detecting the causative organisms of diarrheal illness [19]. We observed that the GI panel had a positive clinical impact in diagnosing sick patients, thereby aiding in diagnosis, antibiotic choice, and patient management, as the majority of severely ill patients had only bacterial infections. We also noted an association between high CRP and GI panel detection of SIEC or *Salmonella*, potentially guiding physicians toward this organism as a cause of pediatric diarrheal illness.

Our study had some limitations, including the collection of data from one hospital involving a distinct population, which limits generalization. Another limitation was the absence of concurrent transmission by other conventional methods for viral and parasitic organisms.

## Conclusions

A GI panel is a potential method of investigation for children with gastroenteritis. The GI panel aids in diagnosis, rationalization of antibiotic therapy, and patient management, including isolation practice and supportive treatment.
